# Hidden threat lurking in extensive hand hygiene during the Covid-19 pandemic: investigation of sensitizing molecules in gel products by hyphenated chromatography techniques

**DOI:** 10.1007/s00216-023-04714-7

**Published:** 2023-05-16

**Authors:** Tania M. G. Salerno, Emanuela Trovato, Giovanna Cafeo, Federica Vento, Mariosimone Zoccali, Paola Donato, Paola Dugo, Luigi Mondello

**Affiliations:** 1 Department of Chemical, Biological, Pharmaceutical and Environmental Sciences, University of Messina, Viale G. Palatucci, 98168 Messina, Italy; 2 Chromaleont S.R.L., at Department of Chemical, Biological, Pharmaceutical and Environmental Sciences, University of Messina, Viale G. Palatucci, 98168 Messina, Italy; 3 Department of Mathematical and Computer Science, Physical Sciences and Earth Sciences, University of Messina, Viale Ferdinando Stagno d’Alcontres 31, 98166 Messina, Italy; 4 Department of Biomedical, Dental, Morphological and Functional Imaging Sciences, University of Messina, Via Consolare Valeria 1, 98125 Messina, Italy

**Keywords:** *Citrus* essential oils, Cosmetics, Covid-19, Furocoumarins, Hand sanitizers, Photosensitizers

## Abstract

During the Covid-19 pandemic, health agencies worldwide have recommended frequent handwashing and sanitizing. A variety of hand gel products were made available on the market, often with fragrances added to curtail the strong smell of alcohol. Commonly used *Citrus* fragrances contain volatile aroma constituents and non-volatile oxygen heterocyclic compounds (OHCs), consisting mostly of polymethoxyflavones, coumarins, and furocoumarins. The latter have long been investigated for their phototoxic properties, and their safety as cosmetic product ingredients has been debated recurrently. To this concern, twelve commercial *Citrus*-scented products were investigated in this study. An extraction method was optimized for thirty-seven OHC compounds, obtaining absolute mean recovery values in the 73.5–116% range with only few milliliters of solvent consumption. Analysis by ultra-high-pressure liquid chromatography with tandem mass spectrometry detection evidenced that three samples did not conform to the labeling requirements for fragrance allergens (coumarin) laid down by the European Union Regulation on Cosmetic Products. The total furocoumarin (FC) content of the samples investigated was in the 0.003–3.7ppm range, with some noteworthy exceptions. Specifically, in two samples, the total FCs were quantified as 89 and 219 ppm, thus exceeding the safe limits recommended up to a factor of 15. Finally, the consistency of the volatile fingerprint attained by gas chromatography allowed drawing conclusions on the authenticity of the *Citrus* fragrances labeled, and several products did not conform to the information reported on the label concerning the presence of essential oils. Besides the issue of product authenticity, analytical tools and regulatory actions for widespread testing of hand hygiene products are urgent, to protect consumers’ health and safety.

**Graphical Abstract:**

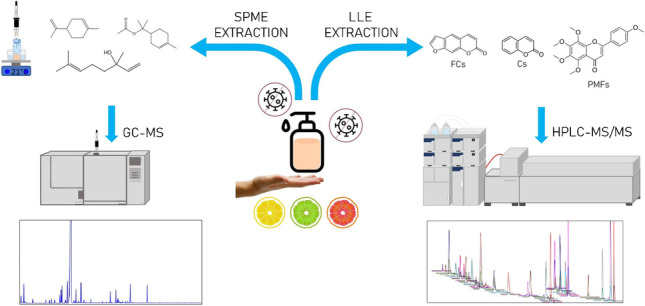

**Supplementary Information:**

The online version contains supplementary material available at 10.1007/s00216-023-04714-7.

## Introduction

In March 2020, the World Health Organization (WHO) declared the Covid-19 outbreak as a pandemic. Containment measures and anti-contagion safety protocols, including social distancing, quarantining, and face mask wearing, were put in place accordingly [1]. The use of hand sanitizers has also been advised as one of the most effective practices to contain the SARS-CoV-2 infection spread. Besides, their use as an alternative to handwashing is expected to remain part of people’s hygiene behavior even after the pandemic era [2]. According to the WHO, an alcohol-based hand sanitizer is “an alcohol-containing preparation (liquid, gel or foam) designed for application to the hands to inactivate microorganisms and/or temporarily suppress their growth…” [3]. The Food and Drug Administration (FDA) regulates hand sanitizers as over-the-counter (OTC) drugs and has released a temporary guidance to expedite their release to market for the duration of the emergency. To comply with the guidelines, compounders must use U.S. Pharmacopoeia–grade ingredients in established proportions, namely isopropyl alcohol (75% v/v) or ethanol (80% v/v), glycerol (1.45% v/v), hydrogen peroxide (0.125% v/v), and sterile distilled water [4]. On the other hand, alcohol-based hand rubs (ABHRs), known as “instant hand sanitizers,” range in alcohol concentrations from 69 to 95% and are available in a variety of delivery formats, such as rinses, gels, and foams. Additional ingredients may be added to the formulations to improve the rheological properties and to increase the acceptability of the ABHR products. Specifically, glycerin, propylene glycol and propanediol are commonly used as humectants to help retain moisture and reduce skin irritation and dryness. Rheology modifiers are also added to deliver the desired formulation esthetics, physical behavior, and flow properties. High molecular weight, cross-linked copolymers like acrylates/C10-30 alkyl acrylate cross polymer or carbomers are typically employed to provide viscosity enhancement and long-term stability. If carbomers are used for thickening, neutralizing with organic bases (i.e., triethanolamine) is required to form water-soluble gels that can tolerate high alcohol concentrations [5]. Finally, fragrances (either natural or synthetic) may be mixed to gelled ABHRs in an attempt to mask unpleasant odors associated with alcohol or other additive ingredients [6].

Gel products for hand hygiene are marketed in the European Union (EU) as biocides or as cosmetic products, such differentiation being based only on the content of active/biocidal ingredients (mainly alcohol). The first type of products fall under the Biocidal Products Regulation BPR (EU) N° 528/2012 [7]. Since these products are intended for a biocidal purpose, their label must include a claim of bacteria/pest mitigation, i.e., “kills 99.9% of virus and bacteria” or “proven activity against enveloped viruses.” Required label declarations also include the type and concentration of the “active ingredient,” the uses for which the product is authorized, warnings, hazard, and precautionary statements.

On the other side, cosmetic hand gels conform to Cosmetic Products Regulation (EC) N° 1223/2009 [8]. Labeling requirements for the marketing authorization include the list of ingredients (in descending order of weight), whereas reporting the concentration of ethanol is not mandatory. Cleaning products are generally considered by regulators not to be intended for a biocidal purpose. Thus, sanitizing properties cannot be claimed in these products’ package, even if antimicrobial ingredients are contained in the formulation. This prevents creating false expectations of consumers on the function of the product.

Cosmetic products for hand hygiene are often scented with synthetic or natural fragrances, such as essential oils (EOs) obtained from different flowers, fruits, or other plant parts [6]. The presence of fragrances may be associated with skin irritation and can cause allergic contact reactions, dermatitis, or photosensitivity. To this regard, the Scientific Committee on Consumer Safety (SCCS) has classified 82 fragrance compounds (among over 3000 used in cosmetic industries) as contact allergens in humans. Out of these, 54 are single chemicals and 28 are natural extracts [9]. However, according to annex III of the Cosmetics Regulation, only 26 fragrance allergens are subject to individual labeling [8–10]. Perfume aromatic compositions and their raw materials are generically referred to as “parfum” or “aroma.”

Among the most valuable fragrances, cold-pressed *Citrus* EOs are widely employed by the cosmetic industry to confer the appreciated top notes to perfumes, soaps, or creams. These olfactory properties are correlated to the presence of volatile organic compounds, mainly terpenes and terpenoids, whereas the non-volatile fraction contributes very little to the *Citrus* aroma. Analyzing both the volatile and non-volatile fractions is useful to identify species-specific patterns and to establish *Citrus* species fingerprinting, being the qualitative and quantitative composition characteristic for each *Citrus* species [11]. The non-volatile portion of *Citrus* EOs is primarily made of oxygen heterocyclic compounds (OHCs); such definition encompasses coumarins (Cs), furocoumarins (FCs), and polymethoxyflavones (PMFs) [12]. The presence of OHCs in *Citrus* has been correlated to various biological effects. There are evidences that PMFs undergo biotransformation in vivo and produce metabolites with pharmacological properties against various disorders [13]. Coumarins make up an important class of phytochemicals ubiquitous in the human diet, and they have been investigated for their anti-inflammatory, anticancer, and antioxidant properties [14]. On the other hand, FCs have been used to treat common inflammatory skin diseases, such as psoriasis and vitiligo [15]. Yet, investigation into the safety of some coumarins used as fixatives or fragrances in cosmetics has identified potential safety concerns of hepatotoxicity, carcinogenicity, and skin sensitization [16]. Psoralens and other FCs are phototoxic and have been positively associated with significantly increased risk of developing cutaneous melanoma [17]. Over the years, a number of different opinions and regulations have been proclaimed by the European Union Regulation and the International Fragrance Association (IFRA), to establish the maximum amount of Cs and FCs in cosmetic products which can be considered safe. The most recent recommendations are summarized in the Supplementary material (Table S1) [8, 18, 19].

Analytical methods relying on high-performance or ultra-high-performance liquid chromatography (HPLC, UHPLC) coupled to UV diode array (PDA) detection are commonly applied for the identification and quantification of furocoumarins. However, these approaches often lack the specificity and sensitivity required [20, 21]. As reviewed by the Analytical Working Group (AWG) of IFRA, HPLC–PDA methods are to be used only in simple cases such as EO analysis. Furthermore, detectable compounds should be present at concentrations higher than 10 mg L^−1^, and peak assignment should be carefully evaluated to avoid misidentification [22]. To this regard, confidence in the identification can be increased by molecular fingerprinting in the mid-IR [23] or by the use of the linear retention indices (LRI) approach [24–26]. On the other hand, hyphenation to tandem mass spectrometry (MS/MS) can provide the sensitivity required for trace-level compounds, to meet the demands for quality control of cosmetics and foods [26, 27].

The research on hand gel products has experienced an exponential increase in the last 3 years, with around 800 articles published on the topic [28]. Among these, only a few have addressed consumers’ safety, specifically focusing on hand-skin conditions, such as skin dryness and eczema [29]. Other studies have investigated the risks of common contaminants found in technical-grade ethanol or methanol used in the formulations [30]. It is noteworthy that these reports have showed that none of the samples analyzed followed WHO recommendations for ABHR/sanitizers [31, 32]. Nevertheless, the amount of furocoumarins and other fragrance ingredients in hand hygiene products has not been evaluated yet. Considering the widespread use of gel products in everyday routine, inspection of the product components becomes important not only for fraud prevention, but primarily to provide the consumer with sufficient information to prevent sensitization or irritation phenomena. Against this backdrop, the present research focused on the characterization of the whole OHC profile of commercially available hand gel/sanitizers scented with *Citrus* EOs (or generically reporting *Citrus* scent on the label). The samples were investigated by means of UHPLC-MS/MS to check the compliance with the limits set by the Regulation, for coumarin and furocoumarin content. Besides, the data obtained for the non-volatile fraction were useful to ascertain the correct labeling for *Citrus* fragrance, in combination with the results of GC-FID and GC–MS analysis of the volatile fraction.

## Materials and methods

### Solvents and standard materials

All the solvents and standard materials were provided by Merck Life Science (Merck KGaA, Darmstadt, Germany), except when otherwise specified**.** Tetrahydrofuran (HPLC grade, purity ≥ 99.9%), water (UHPLC-MS grade, purity ≥ 99.9%), and methanol (LC–MS grade, purity ≥ 99.9%) were used for HPLC–MS/MS analyses. Ethanol (EtOH, gradient grade for HPLC, purity ≥ 99.9%) was used to prepare the stock solutions and to solubilize the extracted materials. Ethyl acetate (HPLC Plus, purity ≥ 99.9%) was used for sample extraction. Acetonitrile (UHPLC-MS, purity ≥ 99.9%) was used to prepare alkyl aryl ketone stock solutions and mixtures.

For GC–MS analyses, a C7-C30 *n*-alkane (1000 mg L^−1^) standard mixture in *n-*hexane was used for the LRI calculation. 4-Nonanol (purity ≥ 96.5%) was used as internal standard at a concentration of 100 mg L^−1^ in EtOH.

For HPLC–MS/MS analyses, the following standard materials were employed: a certified reference mixture of sixteen furocoumarins (250 mg L^−1^ each in acetonitrile), namely 6′,7′-dihydroxybergamottin, 8-geranyloxypsoralen, bergamottin, bergapten, byakangelicin, byakangelicol, epoxybergamottin, heraclenin, imperatorin, isoimperatorin, isopimpinellin, oxypeucedanin, oxypeucedanin hydrate, phellopterin, psoralen, and xanthotoxin and twenty-one compounds from the C, FC, and PMF families, namely 5-geranyloxy-7-methoxycoumarin, 5-O-demethylnobiletin, 8-methoxypsoralen, angelicin, aurapten, citropten, cnidicin, cnidilin, coumarin, epoxyaurapten, gardenin A, gardenin B, herniarin, isomeranzin, meranzin, meranzin hydrate, nobiletin, sinensetin, tangeretin, tetra-O-methylscutellarein, and trioxsalen. Epoxyaurapten reference material was purchased as a powder (> 99% purity) from Labochem (Labochem Science S.r.l, Catania, Italy). For the construction of calibration curves, solutions of the standard materials were prepared in EtOH, in the range from 0.001 to 5.0 mg L^−1^. In detail, working solutions at 5.0, 1.0, 0.5, and 0.1 mg L^−1^ were obtained by diluting a stock multi-analyte solution containing the 37 compounds at 10 mg L^−1^ in EtOH. Further dilutions at 0.01, 0.005, and 0.001 mg L^−1^ were obtained from the 0.1-mg L^−1^ solution. All the standard solutions were stored at − 4 °C, then equilibrated to room temperature and sonicated for 10 min before injection.

Six alkyl aryl ketones in the 8–13 carbon number range (acetophenone, propiophenone, butyrophenone, valerophenone, hexanophenone, heptanophenone) were employed as homolog series for the LRI calculation. The corresponding mixture was prepared at a final concentration of 10 mg L^−1^ each in acetonitrile.

### Samples and sample preparation

Thirteen hydro-alcoholic hand gels were purchased on the internet and at a local supermarket and named progressively from HG-0 to HG-12, as reported in Table 1. All the products contained EtOH as active ingredient, at concentrations ranging between 65 and 75%. Among them, twelve products were selected because of the *Citrus* scent reported in the front labeling. Additionally, in some products, one or more *Citrus* oils were listed among the label ingredients. The only sample purchased as fragrance-free, namely HG-0, was analyzed to confirm the absence of *Citrus* fragrance components, and used as a blank. All the samples were subjected to headspace solid-phase microextraction (HS-SPME) prior to GC analysis, whereas liquid–liquid extraction (LLE) was performed before UHPLC analysis.

**Table 1:** List of the hand gel samples analyzed and their labeled ingredients

Sample	List of ingredients	Front labeling
HG-0	70% alcohol denat., water, PEG-40 hydrogenated castor oil, benzyl alcohol, carbomer, amino methyl propanol, perfume, tocopheryl, acetate, *Aloe barbadensis* leaf juice, citric acid, sodium benzoate	No fragrance
HG-1	65% alcohol denat., water, glycerine, *Aloe barbadensis* leaf oil, allantoin, carbomer, *Citrus aurantium* bergamia oil, lonicera, caprifolium oil, citral, limonene, linalool, triethanolamine	Bergamot
HG-2	70% alcohol denat., water, glycerine, hydroxyethyl cellulose, *Citrus limon* peel oil, *Citrus grandis* (grapefruit) peel oil, *Citrus aurantium* dulcis oil, limonene, citral	Orange, lemon, and grapefruit
HG-3	62% alcohol denat., water, excipients	Lemon
HG-4	77% alcohol denat., water, glycerin, propylene glycol, citric acid, carbomer, amino methyl propanol, isopropyl myristate, tocopheryl acetate, sodium benzoate, denatonium benzoate, parfum, benzyl benzoate, benzyl salicylate, limonene, hexyl cynnamal, linalool, butylphenyl methylpropional	Grapefruit
HG-5	70% alcohol denat	Lemon
HG-6	70% alcohol denat., water, glycerin, parfum (fragrance), acrylates/C10-30 alkyl acrylate crosspolymer, triethanolamine, citral, limonene, linalool, geraniol, eugenol, cinnamal	*Citrus*
HG-7	70% alcohol denat., water, propylene glycol, isopropyl alcohol, carbomer, perfume, triethanolamine, limonene, glycerin, o-phenyl phenol, benzyl benzoate, linalool, citral, geraniol	Lemon
HG-8	70% alcohol denat., *Citrus bergamia* extract, glycerin, hydroethylcellulose, *Citrus bergamia* oil, sodium benzoate, limonene	Bergamot
HG-9	70% alcohol denat., water, glycerin, bergamot essence, natural flavor, E-415, E-131, E-102	Bergamot
HG-10	70% alcohol denat., water, glycerin, PEG-40 hydrogenated castor oil, carbomer, perfume, triethanolamine, limonene, citral, geraniol, eugenol, linalool	Bergamot
HG-11	70% alcohol denat., water, glycerine, propylene glycol, carbomer, triethanolamine, *Melaleuca alternifolia* leaf oil, *Citrus limon* peel oil, citral, limonene	Lemon
HG-12	70% alcohol denat., water, *Citrus bergamia* oil peel expressed, *Citrus limon* peel oil, glycerin, pantenol, perfume, propylene glycol, PEG-40 hydrogenated castor oil, PEG/PPG-25/25 dimethicone, polysorbate 20, carbomer, amino methyl propanol, phenoxyethanol, limonene, linalool, citral	Lemon, bergamot

Genuine cold-pressed bergamot, lemon, and orange EOs were furnished by a local manufacturer (Simone Gatto S.r.l., San Pier Niceto, Italy) and used as a reference for authenticity. All the oil samples were diluted prior to UHPLC analysis, at different ratios, viz. 1:1600, 1:800, and 1:200 for bergamot, lemon, and orange, respectively.

### HS-SPME of the volatile fraction

Three different extraction materials were investigated for method optimization: 1 cm of a polyacrilate 85 μm fiber (PA), 1 cm of a divinylbenzene/carboxen/polydimethylsiloxane (DVB/CAR/PDMS) 50/30 μm fiber, and 1 cm of a polydimethylsiloxane/divinylbenzene (PDMS/DVB) 65 μm fiber (Merck KGaA, Darmstadt, Germany).

The fibers were conditioned before first use according to the manufacturer’s instructions, and a cleaning step of 20 min at 10 °C below the fiber-recommended maximum temperature was applied between consecutive analyses. The extraction procedure was optimized with regard to sample weight (0.2 and 0.5 g), vial volume (10 and 20 mL), sample conditioning time (5 and 10 min), temperature (21 and 40 °C), fiber exposure time (10, 20, and 30 min), and stirring rate (200 and 300 rpm). As a result of the optimization, the maximum yield for volatile extraction was afforded by the PDMS/DVB fiber, under the following experimental conditions: 0.5 g sample weight, 10 mL vial volume, 5 min sample conditioning time, 21 °C temperature, 20 min exposure time, and 300 rpm stirring rate. Following extraction, the analytes were thermally desorbed in the GC injector port for 1 min at 260 °C (splitless mode).

### LLE of the non-volatile fraction

A novel method using water and ethyl acetate was optimized hereby, for the extraction of the non-volatile components of the ABHR samples listed in Table 1. The samples were first shaken vigorously to ensure content homogeneity. Taking also into account the presence of ethanol in the samples, a clear phase separation was obtained only at an EtOH/H_2_O/EtOAc of 1:1.4:1.7 (v/v/v). For most samples containing 70% of ethanol, the following procedure was applied: 1 g of gel was accurately weighed in a glass centrifuge tube, 1.2 mL of water was added, and the mixture was vortexed in an IKA MS 3 basic shaker (IKA-Werke GmbH & Co. KG, Staufen, Germany) at 1500 rpm for 5 min. After the addition of 1.5 mL of ethyl acetate, the mixture was vortexed again at 1500 rpm for 5 min, and centrifuged in a Neya XS centrifuge (REMI Sales & Engineering Ltd, Maharashtra, India) at 3500 rcf for 5 min. Finally, the upper organic layer was collected into a vial and evaporated to dryness using an EZ-2 Series, Genevac rotary evaporator system (Genevac Inc, New York, USA). The solid residue was dissolved in 1 mL of EtOH and sonicated for 30 min, prior to injection in the HPLC–MS/MS system. For samples HG-1, HG-3, HG-4, HG-6, HG-11, and HG-12, the amounts of water and ethyl acetate used as extraction solvents were adjusted according to the different amount of EtOH.

### Instruments and methods

GC–MS analyses were carried out on a Nexis GC-2030 instrument coupled to a GCMS-QP2020 mass spectrometer (Shimadzu Europa, Duisburg, Germany). The separations were achieved on an SLB-5 ms fused-silica capillary column, 30 m × 0.25 mm i.d. × 0.25 μm *df* (Merck KGaA, Darmstadt, Germany). Helium was used as carrier gas at a constant linear velocity of 30.0 cm/s, corresponding to an inlet pressure of 24.2 kPa. The temperature program was as follows: 40 °C for 1 min, to 350 °C at 3 °C/min, held for 5 min. The interface and ion source temperatures were 250 °C and 220 °C, respectively. The acquisition was made in scan mode, in the 40–500 m*/z* range, at a scan rate of 0.2 s. GCMS solution ver. 4.30 software was used for data acquisition and handling (Shimadzu Europa, Duisburg, Germany). For compound identification, the W11N17 (Wiley11-Nist17, Wiley, Hoboken, USA) and FFNSC 4.0 (Shimadzu Europa, Duisburg, Germany) databases were used. The identification was performed by applying two filters: a spectral similarity > 85% and LRI tolerance of ± 10 LRI units.

GC-FID analyses were carried out on a GC-2010 instrument (Shimadzu Europa, Duisburg, Germany). The separations were achieved using the same stationary phase and experimental conditions as for GC–MS analyses. The FID temperature was set at 280 °C; hydrogen and air flows were 40 mL/min and 400 mL/min, respectively. The sampling rate was 200 ms. Data were collected by LabSolution software ver. 5.92 (Shimadzu Europa, Duisburg, Germany), and the quantitative results were determined as peak area percentages.

UHPLC–MS/MS analyses were carried out on a Nexera X2 UHPLC, coupled to a LCMS-8060 triple quadrupole mass spectrometer through an atmospheric pressure chemical ionization (APCI) interface operated in positive polarity (Shimadzu Europa, Duisburg, Germany). The chromatographic system consisted of two LC-30AD pumps, a SIL-30AC autosampler, a DGU-20A5R degassing unit, and a CTO-20AC column oven. The separations were achieved on an Ascentis Express C18 column, 50 × 4.6 mm, 2.7 µm (Merck KGaA, Darmstadt, Germany) at 40 °C. Analyses were performed by  injecting 2 µL of sample. Pumps were set in gradient mode, at a flow rate of 2 mL min^–1^, using water/methanol/THF (85:10:5, *v/v/v*) as mobile phase (A) and methanol/THF (95:5, *v/v*) as mobile phase (B). The gradient program was as follows: 0–4.5 min, 15–28% B; 4.5–7.0 min, 28–60% B; 7.0–11.0 min, 60–85% B, and 11.0–14.0, 85% B. The column was re-conditioned for 5 min after each gradient, resulting in a total analysis time of 19 min.

MS parameters were set as follows: interface temperature, 450 °C; desolvation line temperature, 300 °C; heat block temperature, 300 °C; drying gas flow, 15 L min^–1^; heat gas flow, 3 L min^–1^; and collision gas, 270 kPa. Compounds of the alkyl aryl ketone homologous series were analyzed in single ion monitoring mode (SIM), and specifically acetophenone at *m/z* 121, propiophenone at *m/z* 135, butyrophenone at *m/z* 149, valerophenone at *m/z* 163, hexanophenone at *m/z* 177, and heptanophenone at *m/z* 191. Compounds of the C, FC, and PMF families were analyzed in the multiple ion monitoring mode (MRM) acquisition mode. The MRM parameters (Q, quantifier ion; q, qualifier ion; CE, collision energy; Q1 and Q3 pre-bias) were optimized by direct injection of 2 μL of the standard compounds (10 mg L^−1^) using an isocratic flow of 50% (B), at 0.2 mL min^−1^. The results of MRM optimization are detailed in the Supplementary material (Table S2). For compound identification, an internal MS/MS library was searched by applying two filters: a spectral similarity > 85% and LRI tolerance of ± 4 LRI units [26].

### UHPLC-MS/MS method validation and quantitative data analysis

Quantitative analyses were carried out using external calibration, by calibration curves obtained in the MRM mode for each target analyte in the 0.001–5.0 mg L^−1^ range (7 concentration levels, five replicates for each level). Figures of merit, i.e., limit of detection (LOD), limit of quantification (LOQ), linearity, accuracy and precision were determined according to the Eurachem guidelines [33]. Repeatability was evaluated intra-day (n = 5) and inter-day (3 days, n = 15) as the coefficient of variation (CV%) of the peak areas, calculated at 0.005 mg L^−1^, 0.05 mg L^−1^, and 1 mg L^−1^. Apparent extraction recovery (*R*%) was calculated by adding known amounts (0.005 mg L^−1^, 0.05 mg L^−1^**,** and 1 mg L^−1^) of a multi-analyte solution to the blank sample prior to extraction, over ten measurements. Matrix effects were evaluated by spiking known amounts (0.05 mg L^−1^ and 0.5 mg L^−1^) of the 37-analyte standard mixture to the blank sample after extraction [34]. The peak areas were compared to those obtained for the multi-analyte solution in EtOH. The slopes of the two calibration curves were in good agreement, with slope ratios in the 0.88–1.14 range. For the construction of the calibration curves, regression parameters (i.e., slope, intercept significance, *R*^2^) were evaluated using the function of Microsoft Excel linear regression. The statistical significance for the *y*-intercept was assessed assuming a 95% confidence interval (*α* = 0.05), and the curves were forced to zero for significance values (*p* values) > 0.05. To establish the working linear range, points with a CV% higher than 20 were excluded; moreover, the quartile method was used to detect outliers and to exclude concentration levels outside the lower and upper fences [33]. Linear calibration curves were built by using the unweighted linear regression model (*w* = 1). The increase of the variance across the calibration range (data heteroscedasticity) was evaluated afterwards, by applying an *F*-test and also by plotting of residuals vs concentrations.

Homoscedasticity was not met for any of the 37 analytes investigated, and thus, weighted least square linear regression (WLSLR) was used, applying a *w* = 1/ x^2^  weighting factor [35, 36]. The results of method validation are reported in detail as Supplementary material (UHPLC-MS/MS method validation, Figure S1, and Table S3).

## Results and discussion

Twelve hydro-alcoholic hand gels were investigated, for the declared presence of at least one *Citrus* fragrance among the list of ingredients (or in the front of the package labeling). The samples are listed in Table 1, namely from HG-1 to HG-12. In addition, sample HG-0 was used as a blank, for the absence of *Citrus*-related compounds (fragrance-free). A mislabeling could be noticed straightforward, since samples HG-3 and HG-5 were labeled as “lemon” (front of package label), yet no fragrance ingredients were listed. Moreover, the list of ingredients for samples HG-1 and HG-2 was incorrect, since the botanical names reported did not correspond to any *Citrus* EOs, namely *Citrus aurantium* bergamia (HG-1), *Citrus aurantium dulcis* oil (HG-2), and *Citrus grandis* (grapefruit) peel oil (HG-2). Based on the results obtained by GC- and LC–MS, the presence of bergamot oil (*Citrus bergamia* Risso) could be assumed in sample HG-1, and the presence of sweet orange oil (*Citrus sinensis* (L.), Osbeck) in sample HG-2. Whereas the botanical name *Citrus grandis* identifies pummelo (or shaddock) fruit, the botanical name for grapefruit is *Citrus paradisi* Macfad [37].

Noticeably in sample HG-4, butylphenyl methylpropional was among the labeled ingredients. This synthetic aromatic aldehyde compound, commonly known as “lilial,” has been classified by the EU’s European Commission as a “reprotoxic,” a chemical that adversely affects fertility and fetal development, ruling it “cannot be considered as safe.” Thus, the EU set the March 1, 2020, deadline for all cosmetics with lilial to be pulled off store shelves [38].

### Analysis of the volatile fraction

The sample volatile fraction was investigated by means of HS-SPME followed by GC–MS and GC-FID analysis. The list of compounds identified, and the quantitative results of GC-FID analysis, expressed as percentage peak areas (mean of three replicates ± SD) of the volatile fraction sampled are illustrated in the Supplementary material (Table S4). Only the most illustrative findings are summarized below, concerning the presence of characteristic *Citrus* compounds. The volatile profile obtained for sample HG-0 (fragrance-free) was consistent with the label ingredients. None of the characteristic components of Citrus EOs were detected, while benzyl alcohol and diethyl acetal accounted for 54.24% and 13.22% of the volatiles, respectively. Hereinafter, the data obtained will be discussed according to the *Citrus* fragrance reported in the front labeling of the twelve hand gel samples. Front labels of samples HG-1, HG-8, HG-9, HG-10, and HG-12 reported the claim of bergamot scent. In detail, *Citrus aurantium* bergamia was among the listed ingredients in sample HG-1, together with citral, limonene, and linalool. The typical bergamot constituents were found in the volatile fraction of this sample, i.e., limonene (50.80%), linalool (2.23%), and linalyl acetate (2.56%). As for the citral content, only the *E*-isomer geranial was found (0.10%) among the volatile components. Noticeably, the presence of δ-3-carene and valencene among the volatile components of sample HG-1 suggested the addition of sweet orange EO, not declared in the label [37]. In sample HG-8, *Citrus bergamia* oil and extract were labeled as ingredients, together with limonene. Also in this case, the results of GC analysis (31.33% limonene, 3.47% linalool, and 12.41% linalyl acetate) were in accordance with the literature data on bergamot EO [27]. The only presence of bergamot essence was declared in sample HG-9, and accordingly limonene (39.04%), linalyl acetate (7.41%), and linalool (2.64%) were found in the volatile fraction. The labeled ingredients in sample HG-10 were citral, eugenol, geranial, limonene, linalool, and, generically, perfume. The volatile profile obtained for this sample was compatible with the addition of bergamot fragrance, for the presence of limonene (13.27%), linalool (0.37%), and linalyl acetate (3.95%) [27]. Among the other labeled ingredients, geranial and eugenol were found (0.51% and 0.07%, respectively).

The front label of sample HG-12 reported the presence of bergamot and lemon. *Citrus bergamia* and *Citrus limon* oils were among the listed ingredients, together with citral, limonene, and linalool. Indeed, apart from limonene (33.50%) and linalool (0.26%) listed among the product ingredients, also the presence of linalyl acetate (1.20%) was revealed, which is characteristic of bergamot EO [37]. As for the citral content, only the *E*-isomer geranial was found (0.09%) among the volatile components. In accordance with previous results obtained for *Citrus limon* oils, also the characteristic lemon terpene constituents were present, namely, α-pinene (3.15%), β-pinene (7.89%), and γ-terpinene (9.47%) [27].

The addition of lemon fragrance was claimed in the front labeling of samples HG-3, HG-5, HG-7, and HG-11. However, only in sample HG-11 was *Citrus limon* labeled as an ingredient, together with citral and limonene, and the volatile profile was compatible with the addition of lemon EO: limonene (32.80%), α-pinene (3.85%), β-pinene (10.56%), and γ-terpinene (15.27%) [37].

The fragrance ingredients were not listed in samples HG-3 and HG-5 labels, as discussed earlier. Limonene (64.04%) was the most abundant volatile in sample HG-3. In addition, the presence of α-pinene (0.50%), β-pinene (0.98%), γ-terpinene (1.56%), neral (1.30%), and geranial (1.61%) was compatible with the presence of lemon derivative. Similarly, limonene was the most abundant compound detected in sample HG-5. Moreover, the presence of octanal (0.27%), δ-3-carene (0.45%), and valencene (0.06%) was compatible with the addition of sweet orange derivative (not compliant to the label) [37]. In sample HG-7, limonene was listed among the ingredients, together with citral, geraniol, and linalool. The results of GC analysis (limonene 14.38%, α-pinene 1.76%, β-pinene 1.22%, γ-terpinene 8.39%, neral 0.60%, and geranial 0.79%) were compatible with the presence of lemon derivative.

Sample HG-2 was front labeled for the addition of lemon, orange, and grapefruit fragrances, and *Citrus limon*, *Citrus grandis*, and *Citrus aurantium dulcis* were listed as ingredients, apart from limonene and citral. The composition of the volatile fraction was compatible with the presence of lemon, grapefruit, and sweet orange oils. In detail, the typical *Citrus* constituents found were α-pinene (3.13%), β-pinene (10.00%), δ-3-carene (0.29%), limonene (58.87%), γ-terpinene (8.31%), terpinolene (0.80%), linalool (0.14%), and valencene (trace amounts) [37]. Moreover, geranial was found (0.10%) as part of the citral isomers.

Sample HG-4 was front labeled as scented with grapefruit. The label claimed the presence of parfum, limonene, and linalool. The results obtained for the volatile components, viz. α-terpinene (2.55%), limonene (34.68%), γ-terpinene (5.19%), terpinolene (5.72%), and linalool (4.19%) were different from what was expected for the addition of grapefruit EO. Indeed, the main constituents of a genuine grapefruit EO were absent in the sample, namely α-pinene, sabinene, and β-myrcene [37]. Remarkably, a banned compound was listed among the sample ingredients, as earlier discussed, commonly known as “lilial.” Accordingly, butylphenyl methylpropional was detected among the volatile constituents (0.31%) [38].

Finally, sample HG-6 was front labeled as scented with *Citrus*, and citral, eugenol, geraniol, limonene, and linalool were listed among the ingredients. The results from GC analysis confirmed the presence of all these compounds (30.35% limonene, 2.53% linalool, 2.23% neral, 3.05% geranial, and 0.16% eugenol), except for geraniol. Furthermore, the presence of octanal (0.32%) and δ-3-carene (0.19%) suggested the possible addition of sweet orange. On the other hand, the detection of α-pinene (0.99%), linalyl acetate (4.34%), neryl acetate (0.43%), and geranyl acetate (1.61%) revealed the possible addition of bergamot derivative [37].

### Analysis of the non-volatile fraction

**Fig. 1:**
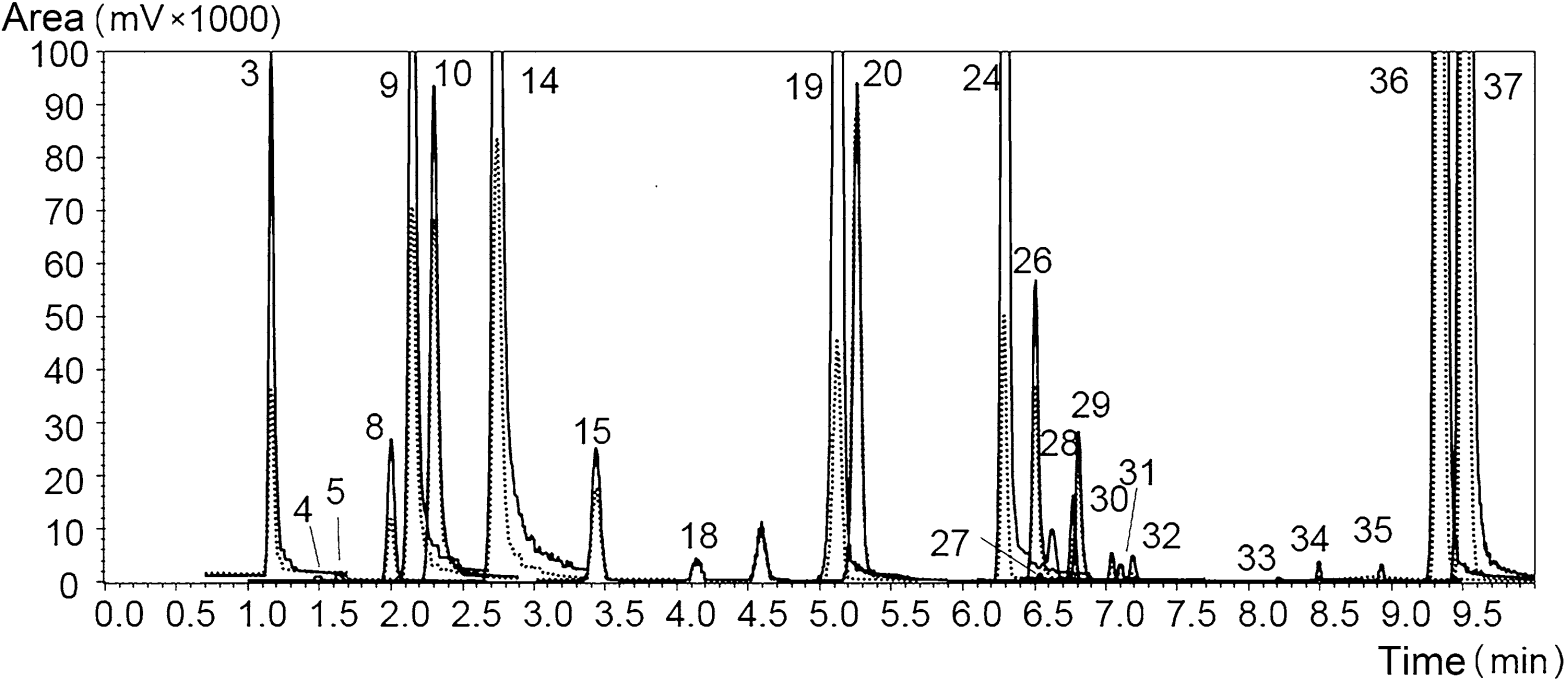
UHPLC-MS/MS (MRM) trace of sample HG-2 (*Q* and *q* transitions in solid and dotted lines, respectively). Peak labeling as in Table 2

The validated UHPLC-MS/MS method was applied to analyze the non-volatile fraction of the thirteen ABHR samples listed in Table 1. Liquid–liquid extraction of the OHC fraction was attained by using water and ethyl acetate as solvents. Samples from HG-0 to HG-10 were injected without further treatment, whereas samples HG-11 and HG-12 were further diluted with EtOH to fit the linearity range (1:10 and 1:100 *v/v*, respectively). Samples were analyzed by injection of 2 μL of each ethanolic extract, and the OHC content was determined by the external calibration method described earlier. By way of example, the UHPLC-MS/MS (MRM) chromatogram of sample HG-2 is reported in Fig. 1. Peak identification was achieved by MRM library search, in combination with an in-house built LRI database [25]. With respect to our previous research, the latter was implemented with coumarin and 6′,7′-dihidroxybergamottin standard compounds, resulting in a total of twenty-one furocoumarins, nine coumarins, and seven polymethoxyflavones included in the method. The results of quantitative evaluation of the 37 target OHC compounds are reported in Table 2.

**Table 2:** Results of the UHPLC-MS/MS determination of oxygen heterocyclic compounds in the ABHR samples listed in Table 1. The target OHC compounds are grouped into chemical families, namely Cs: coumarins, FCs: furocoumarins, and PMFs: polymethoxyflavones. Quantitative results are expressed as micrograms per liter (mean of five replicates and the relative standard deviations)

N°	Name	HG-0	HG-1	HG-2	HG-3	HG-4	HG-5	HG-6	HG-7	HG-8	HG-9	HG-10	HG-11	HG-12
1	Coumarin	< LOQ	2307 ± 9.5	–	–	–	49.2 ± 0.6	78.9 ± 5.7	780.2 ± 16.6	–	–	297.3 ± 2.9	27.4 ± 2.5	–
2	Meranzin hydrate	–	–	–	–	–	–	–	–	–	–	–	–	–
3	Herniarin	–	–	72.5 ± 0.3	–	4.4 ± 0.1	< LOQ	–	–	210.2 ± 7.8	4794 ± 138.3	3.7 ± 0.3	13.7 ± 0.8	32.2 ± 0.7
9	Citropten	–	–	399.6 ± 5.2	2.2 ± 0.2	13.9 ± 0.4	< LOQ	–	–	23,861 ± 710.4	8625 ± 310.8	–	418.1 ± 46.1	498.9 ± 31.8
11	Meranzin	–	–	–	–	–	–	–	–	167.6 ± 13.2	2159 ± 372.3	–	–	–
14	Isomeranzin	–	–	–	–	–	–	–	–	–	4470 ± 423.6	–	–	–
25	Epoxyaurapten	–	–	–	–	–	–	–	–	–	–	–	–	–
35	Aurapten	–	–	1.4 ± 0.1	–	–	–	–	–	22.9 ± 1.6	1020 ± 1.5	–	3.3 ± 0.3	15.5 ± 0.1
37	5-Geranyloxy-7-methoxycoumarin	–	< LOQ	2384 ± 15.9	3.1 ± 0.1	6.4 ± 0.1	8.1 ± 0.1	0.9 ± 0.1	–	10,128 ± 176.2	3111 ± 140.7	3.8 ± 0.1	390.1 ± 34.2	415.5 ± 5.1
**TOT Cs**		** < LOQ**	**2307 ± 9.5**	**2858 ± 54.6**	**5.3 ± 0.4**	**24.7 ± 0.6**	**57.3 ± 0.8**	**79.8 ± 0.6**	**780.2 ± 16.6**	**34,389 ± 909.2**	**24,179 ± 134.9**	**304.8 ± 3.3**	**852.6 ± 278.1**	**962.0 ± 123.3**
4	Biakangelicin	–	–	1.9 ± 0.2	–	–	–	–	–	–	–	–	84.6 ± 4.7	35.1 ± 0.5
5	8-Methoxypsoralen	–	–	< LOQ	–	–	–	–	–	–	–	–	–	–
6	Psoralen	–	–	–	–	–	–	–	–	–	633.5 ± 2.3	–	–	–
7	Angelicin	–	–	–	–	–	–	–	–	–	–	–	–	–
8	Oxypeucedanin hydrate	–	–	35.2 ± 1.3	–	< LOQ	–	–	–	–	–	–	132.2 ± 11.2	70.2 ± 1.9
10	Isopimpinellin	–	–	121.8 ± 2.4	–	–	0.8 ± 0.2	–	–	0.4 ± 0.1	515.2 ± 13.2	–	14.8 ± 1.3	13.6 ± 1.1
12	Heraclenin	–	–	–	–	–	–	–	–	–	–	–	–	2.4 ± 0.3
13	Bergapten	–	< LOQ	180.1 ± 2.0	< LOQ	6.6 ± 0.2	0.8 ± 0.1	< LOQ	< LOQ	21,824 ± 28.5	10,915 ± 156.0	< LOQ	18.4 ± 1.3	300.1 ± 1.1
16	Isobergapten	–	–	–	–	–	–	–	–	–	–	–	–	–
17	Byakangelicol	–	–	–	–	–	–	–	–	–	–	–	18.3 ± 3.7	22.4 ± 2.4
18	Oxypeucedanin	–	–	5.4 ± 0.1	–	–	–	–	–	–	5.7 ± 0.8	–	14.9 ± 1.5	21.4 ± 0.7
21	6′,7′-Dihydroxybergamottin	–	–	< LOQ	–	–	–	–	–	–	–	–	–	0.6 ± 0.1
22	Trioxsalen	–	–	–	–	–	–	–	–	–	–	–	–	–
23	Imperatorin	–	–	–	–	–	–	–	–	–	–	–	2.0 ± 0.7	–
27	Phellopterin	–	–	< LOQ	–	–	–	–	–	–	–	–	26.9 ± 3.4	8.4 ± 0.7
28	Cnidilin	–	–	3.9 ± 0.2	–	–	–	–	–	–	–	–	–	< LOQ
30	Isoimperatorin	–	–	5.7 ± 0.3	–	–	–	–	–	–	63.8 ± 0.4	–	–	–
31	Epoxybergamottin	–	–	4.3 ± 0.4	–	–	10.6 ± 0.6	< LOQ	–	365.9 ± 24.2	4557 ± 81.2	–	–	6.9 ± 0.4
33	Cnidicin	–	–	< LOQ	–	–	–	–	–	–	–	–	4.5 ± 0.6	2.2 ± 0.4
34	8-Geranyloxypsoralen	–	–	108.2 ± 5.1	–	–	–	–	–	–	–	–	221.8 ± 24.0	284.1 ± 13.9
36	Bergamottin	–	< LOQ	1890 ± 7.4	3.8 ± 0.1	16.0 ± 0.2	5.7 ± 0.1	3.1 ± 0.2	–	197,085 ± 8605	72,468 ± 2819	7.9 ± 0.5	425.5 ± 33.7	2892 ± 23.1
**TOT FCs**		–	** < LOQ**	**2357 ± 19.5**	**3.8 ± 0.1**	**22.6 ± 0.4**	**17.9 ± 0.9**	**3.1 ± 0.2**	–	**219,359 ± 8794**	**89,158 ± 3.105**	**7.9 ± 0.5**	**963.7 ± 86.1**	**3659 ± 46.4**
15	Sinensetin	–	–	39.4 ± 1.0	–	–	21.1 ± 0.2	10.5 ± 0.6	–	210.6 ± 10.7	3665 ± 33.9	< LOQ	–	4.0 ± 0.3
19	Nobiletin	–	< LOQ	510.5 ± 25.4	–	–	583.2 ± 1.9	170.1 ± 7.9	–	152.0 ± 3.7	1696 ± 27.2	20.6 ± 0.5	4.1 ± 0.8	3.1 ± 0.3
20	Tetra-O-methylscutellarein	–	–	116.1 ± 1.1	–	–	109.9 ± 1.9	32.8 ± 0.6	–	71.0 ± 5.4	1063 ± 7.7	1.8 ± 0.1	< LOQ	0.5 ± 0.1
24	Tangeretin	–	0.5 ± 0.1	219.5 ± 5.3	0.4 ± 0.1	–	277.7 ± 2.4	60.7 ± 0.8	–	3.5 ± 0.9	65.7 ± 2.3	10.0 ± 0.6	2.0 ± 0.3	0.9 ± 0.1
26	5-O-Demethylnobiletin	–	–	40.0 ± 0.1	< LOQ	–	57.8 ± 2.9	8.0 ± 0.7	–	222.2 ± 21.0	2178 ± 60.7	1.5 ± 0.1	0.4 ± 0.1	2.4 ± 0.3
29	Gardenin A	–	–	15.7 ± 0.7	–	–	33.6 ± 0.6	5.7 ± 0.5	–	–	10.1 ± 1.9	0.8 ± 0.2	–	–
32	Gardenin B	–	–	< LOQ	–	–	–	–	–	27.1 ± 2.6	4.8 ± 0.4	–	–	–
**TOT PMFs**		–	**0.5 ± 0.1**	**941.3 ± 14.2**	**0.4 ± 0.1**	**–**	**1083.4 ± 25.4**	**287.9 ± 23.4**	**–**	**686.3 ± 44.3**	**8682 ± 139.7**	**34.7 ± 1.5**	**6.6 ± 1.2**	**11.1 ± 1.1**
**TOT OHCs**		–	**2308 ± 9.6**	**6156 ± 88.3**	**9.6 ± 1.5**	**47.3 ± 1.0**	**1158.6 ± 26.4**	**370.8 ± 29.7**	**780.2 ± 16.6**	**254,355 ± 1042**	**122,020 ± 316.5**	**347.4 ± 5.3**	**1823 ± 171.2**	**4632 ± 170.8**

Total amounts of Cs, FCs, PMFs and OHCs are reported in bold

Noticeably, the IFRA 50^th^ Amendments [9] sets limits for the presence of certain fragrance ingredients (individually or in combination) in hand gel products (Category 5C, “leave-on” products). Specifically, the total amount of 5-MOP (5-methoxypsoralen, bergapten) used as a marker for furocoumarins should not exceed 15 ppm. For the same category products, a limit of 0.16% is set for 1-benzopyran-2-one (coumarin). Coumarin is also listed in the European Cosmetic Regulation among the 26 fragrance allergens, for which labeling is mandatory for amounts exceeding 0.001% and 0.01% (or 10 and 100 ppm) in leave-on and in rinse-off cosmetic products, respectively [8]. For lower coumarin contents, “perfume” or “aroma” can be generically listed among the sample ingredients.

To this regard, attention was first focused on the amounts of sample coumarin (1-benzopyran-2-one) and furocoumarins, to verify the compliance with the limits laid down by the regulation. Sample HG-0 was first analyzed and selected as blank for the absence of OHC compounds.

As can be seen in Table 2, the coumarin content did not exceed the limits for mandatory labeling set by the EU Regulation, in any of the samples analyzed. In detail, coumarin was not detected within the Cs class in samples HG-2, HG-3, HG-4, HG-8, HG-9, and HG-12, whereas the content was < LOQ in sample HG-0. In the other samples, namely HG-1, HG-5, HG-6, HG-7, HG-10, and HG-11, the coumarin content was in the 0.0274–2.3 ppm range. However, samples HG-1, HG-5, and HG-11 did not conform to the labeling requirements, since no “perfume” or “aroma” was declared among the product ingredients [8]. In most samples analyzed, FCs were quantified at much smaller amounts than those recommended by IFRA for hand gel products [9]. In detail, total FCs were below the LOQ in sample HG-1, and were quantified in the 0.003–3.7 ppm range in the other samples. Samples HG-8 and HG-9 were noteworthy exceptions, in that the total FC amount highly exceeded the safe limits recommended, by a factor of 15 and 6, respectively. Specifically, the total FC amount was around 220 ppm in sample HG-8 and 90 ppm in sample HG-9. Furthermore, some product labels declared *Citrus* EOs as ingredients (Table 1), and thus, their OHC fingerprint was evaluated, to check for consistency with the specific fragrance. To this purpose, the sample PMF constituents were evaluated, as well as authenticity markers.

Front labels of samples HG-1, HG-8, HG-9, HG-10, and HG-12 reported bergamot essence, and *Citrus bergamia* was among the list of ingredients, except for sample HG-10. Qualitatively, the OHC profile of samples HG-1, HG-8, and HG-12 was consistent with that of the genuine cold-pressed bergamot EO, and also in agreement with the literature data [25, 37]. Nonetheless, in sample HG-1, the most abundant constituents of bergamot EO, namely bergamottin, bergapten, and 5-geranyloxy-7-methoxycoumarin, were < LOQ. Noticeably citropten, which also accounts for a significant amount of genuine bergamot EOs, was absent in sample HG-1. It could be concluded that either a small amount of bergamot oil was used in the formulation, or distilled oil was employed. Actually, the presence of OHC compounds in distilled oil is substantially reduced with respect to cold-pressed oil, because of their nonvolatile nature [39]. Furthermore, the presence of tangeretin (0.0005 ppm) and nobiletin (< LOQ) among the PMFs suggested the addition of sweet orange EO (not listed as ingredient), in accordance with the findings of GC-FID and GC–MS analysis.

In sample HG-9, bergamot essence and natural flavor were labeled as ingredients. Accordingly, bergamottin, bergapten, citropten, and 5-geranyloxy-7-methoxycoumarin were quantified among the most abundant OHCs, in the 3–72 ppm range. In addition, typical OHC constituents of *Citrus* oils were found in the non-volatile fraction, namely 5-O-demethylnobiletin, epoxybergamottin, herniarin, isomeranzin, meranzin, nobiletin, sinensetin, and tangeretin [37].

Sample HG-10 front label claimed bergamot scent, while the presence of “perfume” was declared, generically, in the list of ingredients. The sample OHC profile determined by UHPLC-MS/MS did not match that of a bergamot cold-pressed EO, specifically for the absence of citropten and bergapten (< LOQ) within the C and FC class, respectively. Furthermore, the content ratio of bergamottin (compound 36 in Table 2, 0.0079 ppm) and 5-geranyloxy-7-methoxycoumarin (compound 37 in Table 2, 0.0038 ppm) was around 2:1 in sample HG-10. Such value is by far lower than the > 10:1 ratio reported in the literature [25, 37] and hereby determined in the genuine cold-pressed bergamot EO (see Supplementary material, Figure S2). On the other hand, the sample profile is compatible with a “bergapten-free” bergamot oil.

Indeed, due to the phototoxicity of this molecule, bergapten-free oils are obtained with different methods, and marketed. Different from distillation, which reduces all the OHC components, alkaline hydrolysis only affects bergapten and citropten content, while bergamottin and 5-geranyloxy-7-methoxycoumarin rings remain protected by steric hindrance [40, 41]. The front label of sample HG-12 reported the presence of bergamot and lemon: furthermore, *Citrus bergamia* and *Citrus limon* oils were among the listed ingredients. From the results of UHPLC-MS/MS analysis (Table 2), the OHC profile was consistent with the genuine cold-pressed EOs. In detail, bergamottin, citropten, 5-geranyloxy-7-methoxycoumarin, bergapten, 8-geranyloxypsoralen, biakangelicin, herniarin, and oxypeucedanin were determined, in order of abundance.

The addition of lemon fragrance was further claimed in front labeling of samples HG-3, HG-5, HG-7, and HG-11. Sample HG-11 also reported *Citrus limon* peel oil among the ingredients, and its main OHC components were consistent with the genuine cold-pressed EO: bergamottin, citropten, 5-geranyloxy-7-methoxycoumarin, 8-geranyloxypsoralen, oxypeucedanin, and biakangelicin (in order of abundance). As already discussed in the previous paragraph, samples HG-3 and HG-5 were examples of insufficient labeling, since no fragrance ingredients were listed to account for the lemon scent. For sample HG-3, only small amounts of OHCs, namely bergapten, bergamottin, citropten, 5-geranyloxy-7-methoxycoumarin, and tangeretin, were found. These findings suggested the addition of a lemon derivative, in accordance with the results of GC analysis.

**Fig. 2:**
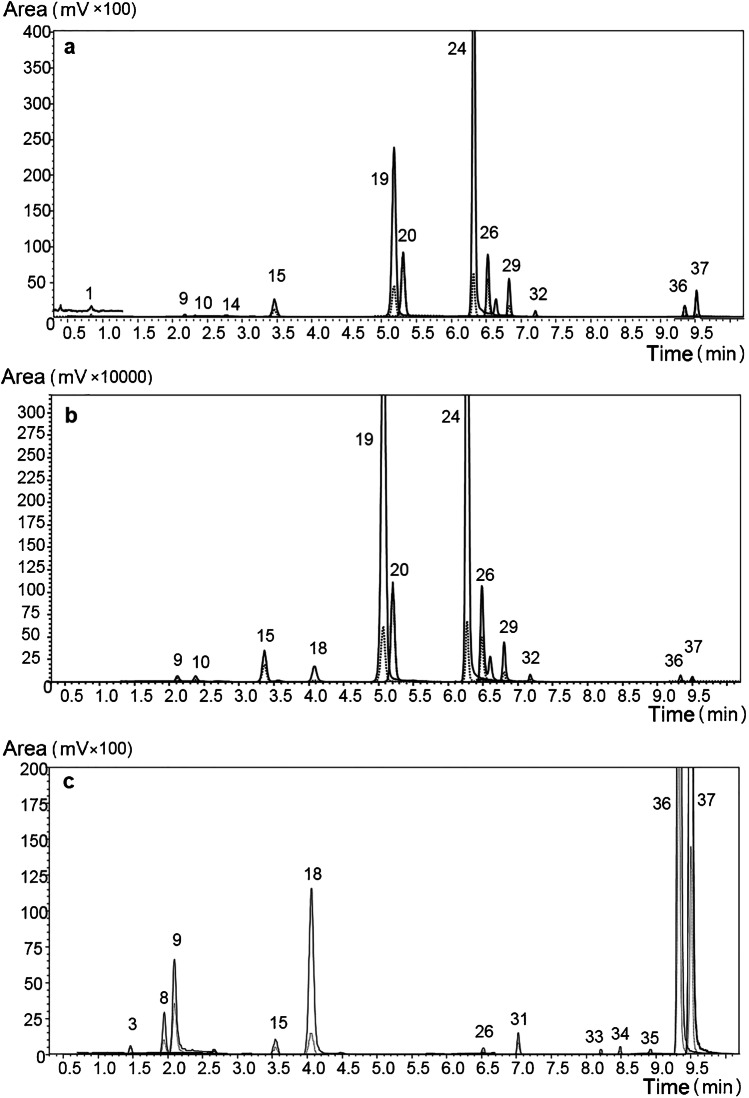
UHPLC-MS/MS (MRM) traces of sample HG-5 (**a**), genuine cold-pressed sweet orange oil (**b**), and genuine cold-pressed lemon oil (**c**). *Q* and *q* transitions are reported in solid and dotted lines, respectively. Peak labeling as in Table 2

On the other hand, the volatile fraction of sample HG-5 was similar to the genuine orange EO (cold-pressed), as can be appreciated in Fig. 2. The UHPLC-MS/MS chromatograms in Fig. 2a, b are in fact superimposable for peaks 19, 20, 24, 26, 29, and 32. The latter were assigned to the following PMF compounds: nobiletin, tetra-O-methylscutellarein, tangeretin, 5-O-demethylnobiletin, gardenin A, and gardenin B. Notably, the same peaks were absent in the UHPLC-MS/MS chromatogram of a genuine cold-pressed lemon EO (Fig. 2c). Such evidence was in accordance with the results of GC analyses, suggesting the presence of sweet orange oil in sample HG-5. Moreover, the amounts of bergamottin (peak 36 in Fig. 2) and 5-geranyloxy-7-methoxycoumarin (peak 37 in Fig. 2) could account for the presence of lemon. Perfume was generically listed among sample HG-7 ingredients, also front-labeled as lemon scented. As earlier discussed, coumarin alone accounted for the whole OHC content of the sample.

Sample HG-2 was front-labeled for the addition of lemon, orange, and grapefruit fragrances, and *Citrus limon*, *Citrus grandis*, and *Citrus aurantium* EOs were listed as ingredients. Also, for this sample, the composition in terms of OHCs was consistent with the label. Specifically, among the FC class, biakangelicin, oxypeucedanin hydrate, 8-geranyloxypsoralen, phellopterin, and cnidicin are typical constituents of lemon [37]. Within the same class, 6′7′-dihydroxybergamottin and epoxybergamottin are typical constituents of grapefruit. As for the PMF class, the sample profile was compatible with the presence of sweet orange EOs [25].

Sample HG-4 was front-labeled for the addition of grapefruit, and perfume was among the labeled ingredients. The small amounts of OHCs found in the sample (herniarin, citropten, bergapten, bergamottin, and 5-geranyloxy-7-methoxycoumarin) did not allow making any conclusions regarding the addition of cold-pressed *Citrus* oils.

Finally, sample HG-6 was scented with *Citrus*, and parfum was listed generically, among the ingredients. The OHC profile resulting from UHPLC-MS/MS analysis was consistent with the addition of sweet orange EO, for the presence of nobiletin (0.17 ppm) and tangeretin (0.06 ppm) as main PMFs. In addition, the small amounts of 5-geranyloxy-7-methoxycoumarin (C class) and bergamottin (FC class) were compatible with the addition of (bergapten-free) bergamot oil [37], as also concluded from the GC results.

## Conclusions

The onset of the Covid-19 pandemic has resulted in an exponential growth of the use of ABHRs and hand sanitizers, with two important effects. The great surge in demand has pushed many manufacturers to produce hand hygiene products, sometimes in the absence of adequate facilities or the quality management required. On the other hand, the need for quick scale-up of production has led to a relaxation of the government control. Moreover the shortage of quality-grade ingredients and raw materials may have led to the release of inadequate formulations or adulterated products.

Thus far, extensive safety evaluation of ABHR/sanitizer products has been conducted by regulatory bodies only in few countries. However, all safety evaluations have targeted only alcohol among the ingredients, resulting in the public bans of methanol-containing samples.

The results of UHPLC-MS/MS analysis performed on commercial hand gel products suggest regular monitoring of the quality of marketed ABHR/sanitizers is urgent, considering the current wide use of these products. Indeed, in 2 of the 13 samples investigated of this study, the FC amount highly exceeded the safe limits recommended, up to a factor of 15. Moreover, 3 hand gel samples did not conform to the labeling requirements, since no “perfume” or “aroma” was declared among the product ingredients, despite the presence of coumarin. In view of these findings, widespread testing of ABHRs should also address the issue of phototoxic and sensitizing ingredients, to protect the consumer health and safety.

In addition, the data gathered from UHPLC-MS/MS, GC-FID, and GC–MS allowed drawing some conclusions on the correct labeling of the market products investigated, also with regard to the authenticity of the *Citrus* scent labeled. Among the products investigated, mislabeling was evidenced in 6 samples, either for insufficient/incorrect description of ingredients, or for incorrect *Citrus* nomenclature. Noticeably, GC-FID and GC–MS analysis confirmed the presence of a banned aldehyde ingredient labeled in one sample, namely lilial, considered harmful to fertility.

## Supplementary Information

Below is the link to the electronic supplementary material.

Supplementary file1 (DOCX 274 KB)
