# Supplementary material for: Hidden threat lurking in extensive hand hygiene during the Covid-19 pandemic: investigation of sensitizing molecules in gel products by hyphenated chromatography techniques
Source: Anal Bioanal Chem. 2023 May 16;415(17):3327–40. doi: 10.1007/s00216-023-04714-7 (PMC10186282; doi:10.1007/s00216-023-04714-7)
Supplement: Supplementary file 1 — Supplementary file1 (DOCX 274 KB) [file 216_2023_4714_MOESM1_ESM.docx]

Supplementary information

Hidden threat lurking in extensive hand hygiene during the Covid-19 pandemic: investigation of sensitizing molecules in gel products by hyphenated chromatography techniques

Tania M.G. Salerno^1^, Emanuela Trovato^1^, Giovanna Cafeo^1^, Federica Vento^2^, Mariosimone Zoccali^3^, Paola Donato^4^, Paola Dugo^1,2^, Luigi Mondello^1,2^*

^1^Department of Chemical, Biological, Pharmaceutical and Environmental Sciences, University of Messina, Viale G. Palatucci, 98168 Messina, Italy

^2^Chromaleont S.r.l., at Department of Chemical, Biological, Pharmaceutical and Environmental Sciences, University of Messina, Viale G. Palatucci, 98168 Messina, Italy

^3^Department of Mathematical and Computer Science, Physical Sciences and Earth Sciences, University of Messina, Viale Ferdinando Stagno d'Alcontres 31, 98166 Messina, Italy

^4^Department of Biomedical, Dental, Morphological and Functional Imaging Sciences, University of Messina, Via Consolare Valeria 1, 98125 Messina, Italy

*lmondello@unime.it

**Table S1.** List of the recent opinions and regulations setting the maximum amounts of coumarin and furocoumarins in cosmetic products

| Year | Opinion/Regulation | Recommendations |
| --- | --- | --- |
| 2009 | European regulation (EC) N^o^ 1223/2009^8^ | *Annex II* - list of substances prohibited in cosmetic products.  **Claim n° 358**. “Furocoumarins except for normal content in natural essences used. In sun protection and in bronzing products, furocoumarins shall be below 1 mg/kg”.    *Annex III* - list of substances which cosmetic products must not contain except subject to the restrictions laid down.  **Ref. n° 77** Coumarin. “The presence of the substance must be indicated in the list of ingredients referred to in Article 19(1)(g) when its concentration exceeds: 0.001% in leave-on products and 0.01% in rinse-off products”. |
| 2020 | International Fragrance Association (IFRA) - 49^th^ Amendment^18^ | For *Citrus* oils and other furocoumarins containing essential oils, restriction limits in the finished product apply. The maximum amount suggested for different categories* is: 15 ppm (0.0015%) of 5-MOP for C1, C2, C3, C4, C5A, C5B, C5C, C5D, C6, C7B, C8, C10B, C11B.  No restriction applies for C7A, C9, C11A, C12.  The use of coumarin in cosmetic is restricted, the following maximum amount is suggested for different categories: C1, 0.089%; C2, 0.080%; C3, 0.089%, C4, 1.5%; C5A, 0.38%; C5B, 0.11%, C5C, 0.16%; C5D, 0.035%; C6, 0.0024%; C7A, 0.18%; C7B, 0.18%; C8, 0.035%; C9, 0.52%; C10A, 0.52%; C10B, 1.6%; C11A, 0.035%; C11B, 0.035%; C12, 33%. |
| 2022 | International Fragrance Association (IFRA) - 50^th^ Amendment^19^ | Same restrictions as in 49^th^ amendment apply for both coumarins and furocoumarins. |

**Table S2.** Optimized MRM parameters for 37 target oxygen heterocyclic compounds. C, coumarin; FC, furocoumarin; PMF, polymethoxyflavone; *Q*, quantifier ion, *q*, qualifier ion; CE, collision energy (V); *Q/q* (%), quantifier-qualifier ratio; LRI, Linear Retention Index.

| ID | Compound | Class | MRM parameters | | | | Window width | | *t*_R_ | LRI |
| --- | --- | --- | --- | --- | --- | --- | --- | --- | --- | --- |
|  |  |  | [M+H]^+^ | Q (CE) | q (CE) | Q/q (%) | Start_(min)_ | End _(min)_ |  |  |
| 1 | Coumarin | C | 147.10 | 91.10 (-22) | 103.10 (-25) | 55 | 0.3 | 1.3 | 0.8 | 774* |
| 2 | Meranzin hydrate | C | 278.90 | 189.15 (-17) | 261.20 (-7.0) | 95 | 0.5 | 1.5 | 1.0 | 786* |
| 3 | Herniarin | C | 177.00 | 121 (-21) | 77.10 (-25) | 37 | 0.7 | 1.7 | 1.2 | 801 |
| 4 | Byakangelicin | FC | 317.0 | 233 (-13) | 231 (-19) | 67 | 1.0 | 2.0 | 1.5 | 825 |
| 5 | 8-methoxypsoralen | FC | 216.90 | 202 (-21) | 174 (-25) | 67 | 1.2 | 2.2 | 1.7 | 836 |
| 6 | Psoralen | FC | 186.90 | 131.10 (-21) | 77.20 (-40) | 71 | 1.3 | 2.3 | 1.7 | 842 |
| 7 | Angelicin | FC | 186.90 | 131.10 (-25) | 77.30 (-35) | 48 | 1.5 | 2.5 | 1.9 | 855 |
| 8 | Oxypeucedanin hydrate | FC | 304.90 | 202.90 (-20) | 147.15 (-32) | 29 | 1.6 | 2.6 | 2.0 | 863 |
| 9 | Citropten | C | 207.00 | 192.05 (-20) | 162.90 (-15) | 61 | 1.7 | 2.7 | 2.2 | 874 |
| 10 | Isopimpinellin | FC | 246.90 | 216.95 (-25) | 232.10 (-18) | 49 | 1.9 | 2.9 | 2.3 | 885 |
| 11 | Meranzin | C | 260.90 | 188.95 (-15) | 131.10 (-29) | 81 | 1.9 | 2.9 | 2.5 | 900 |
| 12 | Heraclenin | FC | 286.90 | 202.90 (-17) | 147.05 (-33) | 30 | 2.3 | 3.3 | 2.7 | 906 |
| 13 | Bergapten | FC | 217.20 | 202.00 (-19) | 174.10 (-25) | 42 | 2.4 | 3.4 | 2.8 | 910 |
| 14 | Isomeranzin | C | 260.90 | 189.25 (-17) | 131.15 (-30) | 95 | 2.2 | 3.2 | 3.0 | 922 |
| 15 | Sinensetin | PMF | 373.00 | 343.00 (-30) | 312.15 (-21) | 57 | 3.0 | 4.0 | 3.5 | 941 |
| 16 | Isobergapten | FC | 217.00 | 201.90 (-21) | 174.20 (-26) | 41 | 3.2 | 4.2 | 3.5 | 942 |
| 17 | Byakangelicol | FC | 316.90 | 218.10 (-29) | 175.05 (-25) | 79 | 3.3 | 4.3 | 3.7 | 950 |
| 18 | Oxypeucedanin | FC | 286.90 | 203.05 (-18) | 59.10 (-38) | 29 | 3.8 | 4.8 | 4.2 | 973 |
| 19 | Nobiletin | PMF | 402.90 | 372.90 (-34) | 327.20 (-31) | 12 | 4.7 | 5.7 | 5.2 | 1022 |
| 20 | Tetra-O-methylscutellarein | PMF | 343.00 | 313.00 (-30) | 282.00 (-25) | 97 | 4.9 | 5.9 | 5.3 | 1030 |
| 21 | 6’-7’-dihydroxybergamottin | FC | 373.40 | 203.05 (-25) | 147.05 (-40) | 37 | 5.5 | 6.5 | 6.0 | 1068 |
| 22 | Trioxsalen | FC | 228.95 | 142.00 (-25) | 173.05 (-22) | 70 | 5.7 | 6.7 | 6.1 | 1074 |
| 23 | Imperatorin | FC | 270.90 | 203.10 (-15) | 147.20 (-31) | 50 | 5.8 | 6.8 | 6.2 | 1082 |
| 24 | Tangeretin | PMF | 372.90 | 342.95 (-30) | 211.20 (-34) | 8 | 5.9 | 6.9 | 6.3 | 1088 |
| 25 | Epoxyaurapten | C | 315.20 | 162.90 (-16) | 107.30 (-25) | 33 | 6.0 | 7.0 | 6.5 | 1097 |
| 26 | 5-O-demethylnobiletin | PMF | 388.90 | 358.95 (-30) | 341.15 (-27) | 34 | 6.1 | 7.1 | 6.5 | 1100 |
| 27 | Phellopterin | FC | 301.10 | 233.15 (-14) | 218.10 (-30) | 34 | 6.1 | 7.1 | 6.6 | 1103 |
| 28 | Cnidilin | FC | 301.00 | 203.90 (-15) | 217.95 (-28) | 24 | 6.4 | 7.4 | 6.8 | 1128 |
| 29 | Gardenin A | PMF | 418.90 | 389.00 (-32) | 371.15 (-28) | 36 | 6.4 | 7.4 | 6.8 | 1132 |
| 30 | Isoimperatorin | FC | 270.90 | 203.008 (-15) | 147.15 (-31) | 47 | 6.6 | 7.6 | 7.1 | 1158 |
| 31 | Epoxybergamottin | FC | 355.20 | 203.10 (-18) | 215.20 (-19) | 23 | 6.7 | 7.7 | 7.1 | 1165 |
| 32 | Gardenin B | PMF | 358.90 | 328.95 (-29) | 311.20 (-25) | 28 | 6.8 | 7.8 | 7.2 | 1174 |
| 33 | Cnidicin | FC | 355.00 | 219.05 (-16) | 172.95 (-32) | 54 | 7.8 | 8.8 | 8.2 | 1302* |
| 34 | 8-geranyloxypsoralen | FC | 338.90 | 202.95 (-25) | 95.10 (-25) | 77 | 8.1 | 9.1 | 8.5 | 1315* |
| 35 | Aurapten | C | 299.00 | 163.00 (-15) | 107.20 (-40) | 27 | 8.5 | 9.5 | 9.0 | 1336* |
| 36 | Bergamottin | FC | 338.90 | 203.10 (-14) | 147.10 (-35) | 23 | 9.00 | 10.0 | 9.4 | 1356* |
| 37 | 5-geranyloxy-7-methoxycoumarin | C | 329.00 | 192.95 (-20) | 149.10 (-25) | 10 | 9.2 | 10.2 | 9.5 | 1364* |

* Values extrapolated from the equation obtained by plotting the carbon number of the LRI standard mixture *vs* the retention times.

UHPLC-MS/MS method validation

The increase of variance across the calibration range (data heteroscedasticity) was evaluated, in order to counteract the greater influence of the higher concentration levels on the fitted regression lines. Heteroscedasticity was tested by applying an F-test (for the homogeneity of variance) and also by plotting of residuals vs concentrations. The F-test was performed by calculating the experimental F value (F_exp_), and comparing it to the F tabulated value (F_tab_) at confidence level of 95%, and n-1 degrees of freedom. Afterwards, the residues were calculated by determining the difference between the measured areas (y observed) and the interpolated value obtained from the regression equation (y predicted) [34,35].

Linear calibration curves were built in EtOH by using the unweighted linear regression model (*w*=1). After performing F-test, homoscedasticity was not met for any of the 37 analytes investigated, with F_exp_ resulting always higher than F_tab_ (6.39; α=0.05; *f_1_, f_2_*= 4 degrees of freedom). Thus, Weighted Least Square Linear Regression (WLSLR) was used to find the best fit. Subsequently, the results of residual calculations were plotted *vs* concentrations. The residual plot obtained for coumarin is shown in Figure S1 as an example.

It can be clearly appreciated that linear regression does not fit equally across the calibration range, hence the use of an adequate weighting factor was necessary. For each analyte investigated, four different weighted regression models were compared (w=1/x^2^, w=1/x, w=1/y^2^ and w=1/y) and the sum of the relative errors was calculated as: %RE = (C_exp_-C_nom_/C_nom_) ×100, where C_exp_ is the experimental concentration obtained from the weighted equation, and C_nom_ is the theoretical concentration. The weighting factors affording the smallest ΣRE% values where chosen to describe the concentration-response relationship, accordingly. The use of WLSR method (using w=1/x2) afforded higher accuracy with respect to the linear least square regression (LLSR) model. For instance, the %bias at the lower end of the calibration range of coumarin was 174 when using LLSR, thus significantly higher than the acceptable limits (+/- 20). After applying the correct weighting factor, the bias was reduced to 8.8, as reported in Table S3. Moreover the WLSR method applying a w=1/x2 weighting factor also afforded the smallest ΣRE% (44.06) compared to the other weighted regression models (69.5, 64.8 and 46.86 for w=1/x, w=1/y2 and w=1/y weighting factors, respectively).


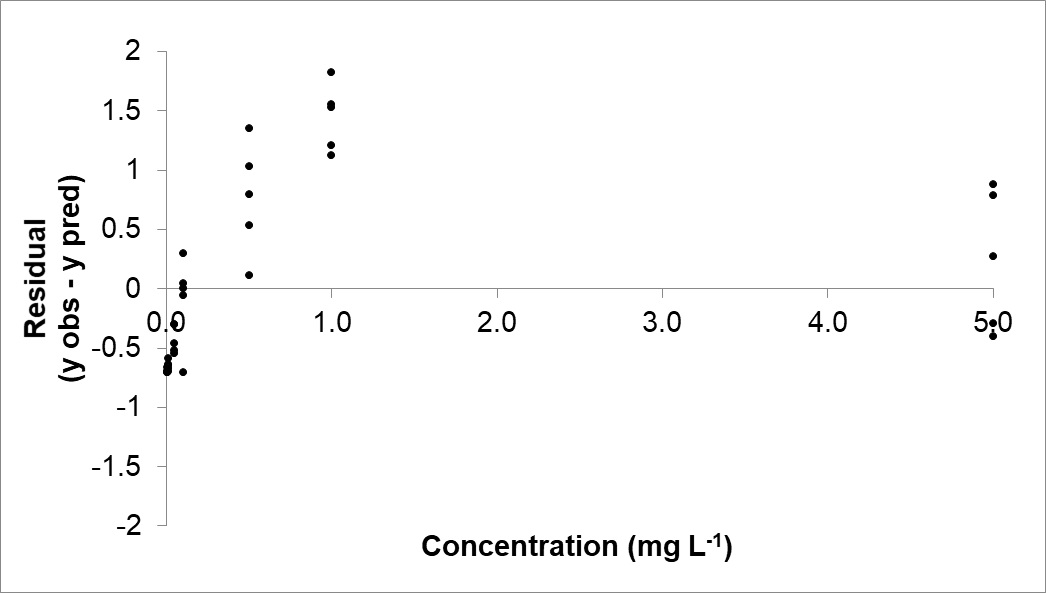


Figure S1. Residual vs. concentration plots for coumarin (data obtained from five intra-day replicates for each calibration level).

**Table S3.** Results of unweighted (w=1, y= 391072x + 6512.4) and weighted (w=1/x2, y= 404821x - 101.5) regression models applied to UHPLC-MS/MS calibration data of coumarin. C_nom_: theoretical concentration, C_exp_: experimental concentration obtained from the weighted equation.

| C_nom_  (mg L^-1^) | C_exp*_  (*w*=1) | C_exp*_  (*w* =1/x^2^) | %BIAS | |
| --- | --- | --- | --- | --- |
|  |  |  | Unweighted  (*w*=1) | Weighted  (*w* =1/x^2^) |
| 0.010 | -0.007 | 0.009 | 174.75 | 8.84 |
| 0.050 | 0.038 | 0.053 | 24.23 | 5.87 |
| 0.100 | 0.089 | 0.102 | 11.24 | 2.08 |
| 0.500 | 0.513 | 0.512 | 2.64 | 2.42 |
| 1.000 | 1.036 | 1.017 | 3.57 | 1.69 |
| 5.000 | 4.992 | 4.839 | 0.16 | 3.23 |

*mean values of five intra-day replicates

**Table S4.** Results of GC-FID analysis of the hand gel samples investigated (listed in Table 1). Relative amounts of the characteristic *Citrus* compounds are expressed as mean ± SD of three replicates. LRI_exp_: experimental Linear Retention Indices calculated on SLB-5ms fused-silica capillary column.

| **ID #** | **Compound** | **LRI_exp_** | **HG-0** | **HG-1** | **HG-2** | **HG-3** | **HG-4** | **HG-5** | **HG-6** | **HG-7** | **HG-8** | **HG-9** | **HG-10** | **HG-11** | **HG-12** |
| --- | --- | --- | --- | --- | --- | --- | --- | --- | --- | --- | --- | --- | --- | --- | --- |
| 1 | Diethyl acetal | 722 | 13.22±0.92 | - | - | - | - | - | - | - | - | - | - | - | - |
| 2 | α-Thujene | 927 | - | 0.04±0.00 | 0.63± 0.01 | 0.04 ± 0.00 | 0.13 ± 0.00 | 0.12 ± 0.00 | 0.03 ± 0.00 | 0.12 ± 0.00 | 1.46 ± 0.02 | 1.01 ± 0.02 | 0.06 ±0.00 | 0.44 ±0.01 | 0.46 ± 0.01 |
| 3 | α-Pinene | 933 | - | 2.33± 0.04 | 3.13± 0.05 | 0.50 ± 0.01 | 1.09 ± 0.02 | 1.47 ± 0.03 | 0.99 ± 0.02 | 1.76 ± 0.03 | 3.41 ± 0.06 | 3.13 ± 0.05 | 1.59 ± 0.03 | 3.85 ± 0.07 | 3.15 ± 0.05 |
| 4 | Benzaldehyde | 960 | 1.39 ± 0.10 | - | - | - | - | - | - | - | - | - | - | - | - |
| 5 | Sabinene | 972 | - | 0.01±0 .00 | 1.76 ± 0.03 | - | - | 0.85 ± 0.01 | - | - | - | - | 0.06 ± 0.00 | - | 0.13 ± 0.00 |
| 6 | β-Pinene | 978 | - | 2.58±0.04 | 10.00 ± 0.17 | 0.98 ± 0.02 | 2.52 ± 0.04 | 0.48 ± 0.01 | 0.09 ± 0.00 | 1.22 ± 0.02 | 10.54 ± 0.18 | 10.51 ±0.18 | 0.83 ± 0.01 | 10.56 ± 0.18 | 7.89 ± 0.13 |
| 7 | Myrcene | 991 | 0.77 ± 0.05 | 7.53±0.13 | 6.17 ± 0.11 | 2.36 ± 0.04 | 1.29 ± 0.02 | 2.97 ± 0.05 | 6.78 ± 0.12 | 0.78 ± 0.01 | 5.58 ± 0.10 | 7.69 ± 0.13 | 1.49 ± 0.03 | 2.29 ± 0.04 | 2.78 ± 0.05 |
| 8 | *n*-Octanal | 1006 | - | - | - | - | 2.58 ± 0.04 | 0.27 ± 0.00 | 0.32 ± 0.01 | 0.20 ± 0.00 | 0.03 ± 0.00 | - | - | - | - |
| 9 | α-Phellandrene | 1007 | - | - | - | 0.59 ± 0.01 | - | - | - | - | - | - | - | 0.57 ± 0.01 | - |
| 10 | δ-3-Carene | 1009 | - | 1.79±0.03 | 0.29 ± 0.00 | 0.44 ± 0.01 | 0.09 ± 0.00 | 0.45 ± 0.01 | 0.19 ± 0.00 | 0.08 ± 0.00 | - | - | 0.06 ± 0.00 | 0.37 ± 0.01 | - |
| 11 | 1,4-Cineole | 1016 | - | - | - | - | - | - | - | 5.41 ± 0.09 | - | - | - | - | - |
| 12 | α-Terpinene | 1018 | - | 0.21±0.00 | 0.15 ± 0.00 | 4.66 ± 0.08 | 2.55 ± 0.04 | - | 0.06 ± 0.00 | 4.67 ± 0.08 | 0.30 ± 0.01 | - | 0.05 ± 0.00 | 5.33 ± 0.09 | 0.76 ± 0.01 |
| 13 | *p*-Cymene | 1025 | - | 0.59±0.03 | - | - | 1.25 ± 0.02 | - | - | 3.57 ± 0.06 | 0.31 ± 0.01 | 0.05 ± 0.00 | 0.32 ± 0.01 | - | - |
| 14 | Limonene | 1030 | - | 50.80±0.88 | 58.87 ± 1.00 | 64.04 ± 1.09 | 34.68 ± 0.59 | 63.74 ± 1.09 | 31.07 ± 0.54 | 14.38 ±0.25 | 31.33 ± 0.54 | 39.04 ± 0.67 | 13.27 ±0.23 | 40.36 ± 0.69 | 33.50 ± 0.57 |
| 15 | (*Z*)-β-Ocimene | 1037 | - | - | - | - | - | - | 0.10 ± 0.00 | - | - | - | 0.34 ± 0.01 | - | 0.16 ± 0.01 |
| 16 | Benzyl alcohol | 1041 | 54.24 ± 3.78 | - | - | - | - | - | - | - | - | - | - | - | - |
| 17 | (*E*)-β-Ocimene | 1046 | 0.79 ± 0.05 | 1.40 ± 0.02 | - | 0.34 ± 0.01 | - | 0.12 ± 0.00 | 0.24 ± 0.01 | 0.46 ± 0.01 | 2.73 ± 0.05 | 2.58 ± 0.04 | 0.66 ± 0.01 | - | 0.76 ± 0.01 |
| 18 | γ-Terpinene | 1058 | - | 1.31 ± 0.02 | 8.31 ± 0.14 | 1.56 ± 0.03 | 5.19 ± 0.09 | 1.30 ± 0.02 | 0.14 ± 0.00 | 8.39 ± 0.14 | 10.15 ± 0.17 | 11.08± .19 | 0.20 ± 0.00 | 15.27 ± 0.26 | 9.47 ± 0.16 |
| 19 | *n*-Octanol | 1076 | - | - | - | - | - | - | - | - | - | - | - | - | - |
| 20 | Terpinolene | 1086 | - | 1.03 ± 0.02 | 0.80 ± 0.01 | 2.32 ± 0.04 | 5.72 ± 0.10 | 6.40 ± 0.11 | 0.31 ± 0.01 | 13.25±0.23 | 1.12 ± 0.02 | 1.80 ± 0.03 | 0.17 ± 0.00 | 3.11 ± 0.05 | 1.32 ± 0.02 |
| 21 | Tetrahydro linalool | 1098 | 3.16 ± 0.22 | - | - | - | - | - | - | - | - | - | - | - | - |
| 22 | Linalool | 1101 | - | 2.23 ± 0.04 | 0.14 ± 0.00 | - | 4.19 ± 0.07 | 0.99 ± 0.02 | 2.53 ± 0.04 | - | 3.47 ± 0.06 | 2.64 ± 0.04 | 0.37 ± 0.01 | 0.14 ± 0.00 | 0.26 ± 0.00 |
| 23 | (4*E*,6*Z*)-allo-Ocimene | 1127 | 0.68 ± 0.05 | - | - | - | - | - | - | - | - | - | - | - | - |
| 24 | Neo-allo-ocimene | 1139 | 0.51 ± 0.03 | - | - | - | - | - | - | - | - | - | - | - | - |
| 25 | Terpinen-4-ol | 1184 | - | - | - | - | - | - | - | - | - | - | - | 2.70 ± 0.05 | - |
| 26 | Gardenol | 1187 | 1.17 ± 0.08 | - | - | - | - | - | - | - | - | - | - | - | - |
| 27 | α-Terpineol | 1196 | - | - | - | 0.19 ± 0.00 | - | 0.30 ± 0.01 | - | 0.68 ± 0.01 | - | - | - | 0.23 ± 0.00 | - |
| 28 | *n*-Decanal | 1208 | - | - | 0.02 ± 0.00 | 0.08 ± 0.00 | - | 0.01 ± 0.00 | - | 0.02 ± 0.00 | - | - | - | 0.02 ± 0.00 | - |
| 29 | Neral | 1238 | - | - | - | 1.30 ± 0.02 | - | 0.01 ± 0.00 | 2.23 ± 0.04 | 0.66 ± 0.01 | - | - | - | - | - |
| 30 | Linalyl acetate | 1250 | - | 2.56 ± 0.04 | - | - | 0.10 ± 0.00 | - | 4.34 ± 0.07 | - | 12.41 ± 0.21 | 7.41 ± 0.13 | 3.95 ± 0.05 | - | 1.20 ± 0.02 |
| 31 | Isopropyl  phenylacetate | 1266 | 0.55 ± 0.04 | - | - | - | - | - | - | - | - | - | - | - | - |
| 32 | Geranial | 1268 | - | 0.10 ± 0.00 | 0.10 ± 0.00 | 1.61 ± 0.03 | - | 0.02 ± 0.00 | 3.05 ± 0.06 | 0.79 ± 0.01 | 0.11 ± 0.00 | 0.10 ± 0.00 | 0.51 ± 0.01 | - | 0.09 ± 0.00 |
| 33 | p-t-Butyl cyclohexyl-acetate (*Z*) | 1332 | 2.94 ± 0.20 | - | - | - | - | - | - | - | - | - | - | - |  |
| 34 | (*E*)-Ethyl-Linalyl  acetate | 1342 | 0.63 ± 0.04 | - | - | - | - | - | - | - | - | - | - | - | - |
| 35 | α-Terpinyl acetate | 1349 | - | 0.19 ± 0.00 | - | 2.45 ± 0.04 | - | - | 0.83 ± 0.01 | 0.20 ± 0.00 | 0.23 ± 0.00 | 0.15 ± 0.00 | 0.02 ± 0.00 | - | 0.01 ± 0.00 |
| 36 | Eugenol | 1351 | - | - | - | - | - | - | 0.16 ± 0.01 | - | - | - | 0.07 ± 0.00 | - | - |
| 37 | Neryl acetate | 1361 | - | 0.27 ± 0.00 | 0.03 ± 0.00 | 0.79 ± 0.01 | - | 0.03 ± 0.00 | 0.43 ± 0.01 | 0.03 ± 0.00 | 1.38 ± 0.02 | 0.86 ± 0.01 | 0.24 ± 0.00 | 0.10 ± 0.00 | 0.15 ± 0.00 |
| 38 | *p-t*-Butyl cyclohexyl-acetate (*E*) | 1368 | 3.27 ± 0.23 | - | - | - | - | - | - | - | - | - | - | - | - |
| 39 | Geranyl acetate | 1380 | - | 0.81 ± 0.01 | 0.01 ± 0.00 | 0.01 ± 0.00 | - | 0.01 ± 0.00 | 1.61 ± 0.03 | 0.05 ± 0.00 | 2.05 ± 0.04 | 1.15 ± 0.02 | 0.43 ± 0.01 | 0.05 ± 0.00 | 0.21 ± 0.00 |
| 40 | (*E*)-Caryophyllene | 1424 | - | - | - | 0.01 ± 0.00 | - | 0.11 ± 0.00 | 3.36 ± 0.06 | 0.89 ± 0.02 | 1.42 ± 0.02 | 1.77 ± 0.03 | - | - | 0.38 ± 0.01 |
| 41 | (*E*)-α-Bergamoptene | 1432 | - | - | - | 0.01 ± 0.00 | - | 0.07 ± 0.00 | 0.01 ± 0.00 | 0.07 ± 0.00 | 1.18 ± 0.02 | 1.60 ± 0.03 | - | - | - |
| 42 | Valencene | 1492 | - | 0.12 ± 0.00 | 0.01 ± 0.00 | - | - | 0.06 ± 0.00 | - | - | - | - | - | 0.04 ± 0.00 | - |
| 43 | (*E*,*E*)-α-Farnesene | 1504 | - | - | - | - | - | - | - | - | - | - | - | - | - |
| 44 | β-Bisabolene | 1508 | - | - | - | - | - | - | 0.11 ± 0.00 | - | 0.60 ± 0.01 | 1.06 ± 0.02 | - | - | 0.15 ± 0.00 |
| 45 | Lilial | 1523 | - | - | - | - | 0.31 ± 0.01 | - | - | - | - | - | - | - | - |
| **Total** | |  | **83.32 ± 2.20** | **75.91 ± 1.29** | **90.42 ± 1.54** | **84.31 ± 1.44** | **61.68 ± 1.06** | **79.79 ± 1.36** | **59.78 ± 1.13** | **57.69 ± 0.98** | **89.82 ± 1.53** | **93.59 ± 1.60** | **24.70 ±0.42** | **85.42 ± 1.46** | **62.67 ± 1.07** |


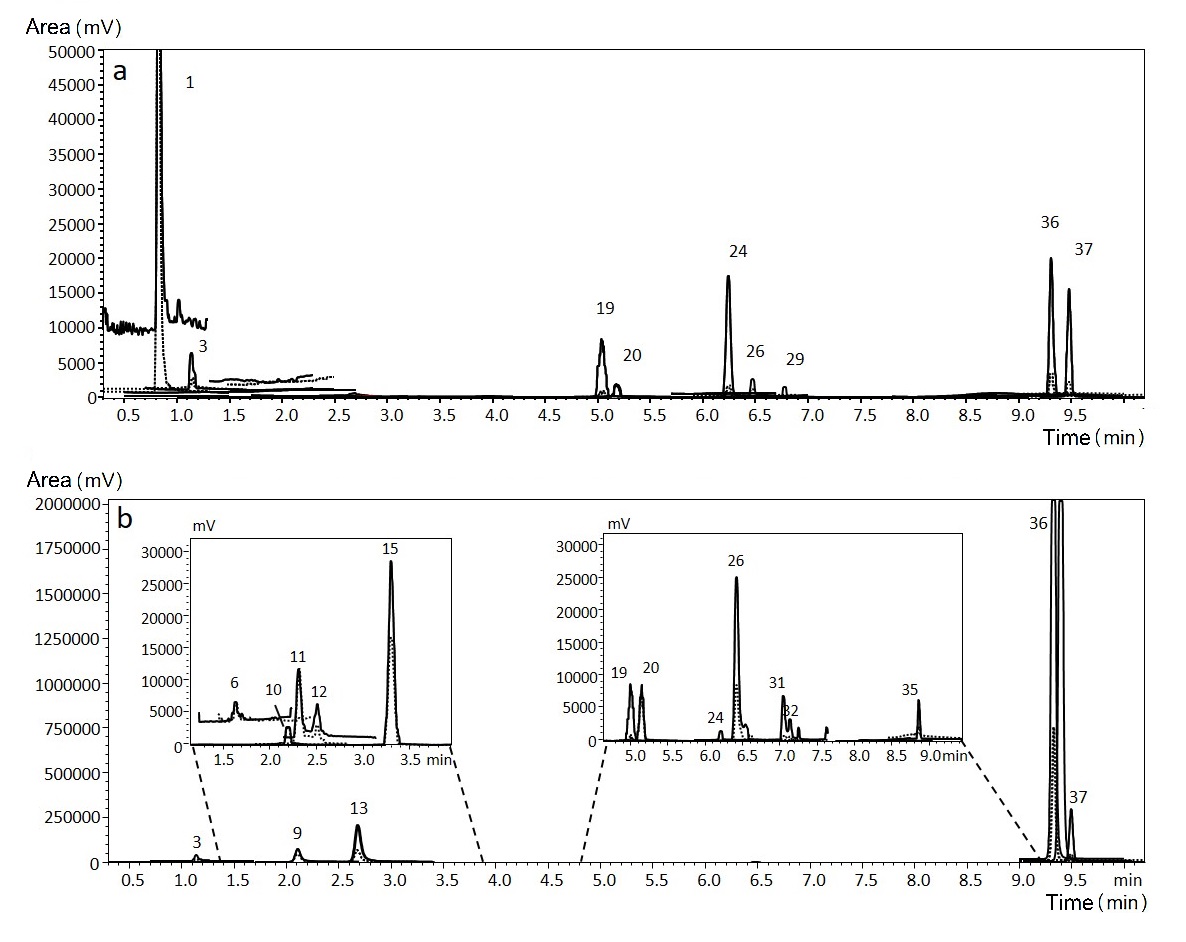


Figure S2. HPLC-MS/MS (MRM) traces of sample HG-10 (a), genuine cold-pressed bergamot oil (b). The insets show low trace compounds in genuine cold-pressed bergamot oil (b). *Q* and *q* transitions are reported in solid and dotted line, respectively. Peak labeling as in Table
